# Identification of secondary targets of N-containing bisphosphonates in mammalian cells via parallel competition analysis of the barcoded yeast deletion collection

**DOI:** 10.1186/gb-2009-10-9-r93

**Published:** 2009-09-10

**Authors:** Nicoletta Bivi, Milena Romanello, Richard Harrison, Ian Clarke, David C Hoyle, Luigi Moro, Fulvia Ortolani, Antonella Bonetti, Franco Quadrifoglio, Gianluca Tell, Daniela Delneri

**Affiliations:** 1 Department of Biomedical Sciences and Technologies, University of Udine, Piazzale Kolbe, 33100, Udine, Italy; 2 Faculty of Life Science, University of Manchester, Oxford Road, M13 9PT, Manchester, UK; 3 School of Biological Sciences, Institute of Evolutionary Biology, King's Buildings, West Mains Road, Edinburgh EH9 3JT, UK; 4 The Center for the Study of Metabolic Bone Diseases, via Vittorio Veneto, 34170, Gorizia, Italy; 5 Department of Medical and Morphological Research, University of Udine, Piazzale Kolbe, 33100, Udine, Italy

## Abstract

**Background:**

Nitrogen-containing bisphosphonates are the elected drugs for the treatment of diseases in which excessive bone resorption occurs, for example, osteoporosis and cancer-induced bone diseases. The only known target of nitrogen-containing bisphosphonates is farnesyl pyrophosphate synthase, which ensures prenylation of prosurvival proteins, such as Ras. However, it is likely that the action of nitrogen-containing bisphosphonates involves additional unknown mechanisms. To identify novel targets of nitrogen-containing bisphosphonates, we used a genome-wide high-throughput screening in which 5,936 *Saccharomyces cerevisiae *heterozygote barcoded mutants were grown competitively in the presence of sub-lethal doses of three nitrogen-containing bisphosphonates (risedronate, alendronate and ibandronate). Strains carrying deletions in genes encoding potential drug targets show a variation of the intensity of their corresponding barcodes on the hybridization array over the time.

**Results:**

With this approach, we identified novel targets of nitrogen-containing bisphosphonates, such as tubulin cofactor B and ASK/DBF4 (Activator of S-phase kinase). The up-regulation of tubulin cofactor B may explain some previously unknown effects of nitrogen-containing bisphosphonates on microtubule dynamics and organization. As nitrogen-containing bisphosphonates induce extensive DNA damage, we also document the role of DBF4 as a key player in nitrogen-containing bisphosphonate-induced cytotoxicity, thus explaining the effects on the cell-cycle.

**Conclusions:**

The dataset obtained from the yeast screen was validated in a mammalian system, allowing the discovery of new biological processes involved in the cellular response to nitrogen-containing bisphosphonates and opening up opportunities for development of new anticancer drugs.

## Abstract 2

Growth competition assays using barcoded yeast deletion-mutants reveal the molecular targets of nitrogen containing bisphosphonates used for the treatment of bone cancers and osteoporosis.

## Background

We exploited the molecular tools available for *Saccharomyces cerevisiae *to investigate potential targets of the nitrogen-containing bisphosphonates (N-BPs) alendronate (ALE), ibandronate (IBA) and risedronate (RIS). N-BPs are pyrophosphate analogs used to treat osteoporosis and, at high doses, cancer-induced bone disease [1]. The primary target of N-BPs is farnesyl pyrophosphate synthase (FPPS), whose inhibition prevents protein prenylation [2,3]. *In vitro *studies conducted on tumor cell lines suggest that N-BPs are able to exert a broad spectrum of actions, including inhibition of invasion, and promotion of cell cycle arrest [1]. However, little is known about the molecular mechanisms underlying these effects. In this context, we performed a large-scale competition experiment with different yeast mutants in the presence of sub-lethal doses of N-BPs to unravel their secondary cellular targets and to understand the molecular changes occurring in cells exposed to such compounds.

The yeast experimental system consists of a collection of 5,936 heterozygote deletant strains encompassing all yeast's open reading frames (ORFs) [4,5]. Each mutant carries two molecular barcodes (TAGs), which are 20-bp unique sequences acting as strain identifiers. The mutants are grown together in competition under different selective pressures, and the molecular TAGs are discriminated on a hybridization array. The strains carrying deletions in genes that are crucial for the yeast growth in the given conditions will loose the competition, scored by a progressively lower intensity of their barcodes on the array over the time. This approach has been successfully used to functionally characterize all yeast ORFs [4,5], to identify human genes involved in mitochondrial diseases [6] and to identify drug targets [4,7-9]. Moreover, genes that are quantitatively important in different environments, so that, when one allele is missing, the resulting phenotype is either severely compromised (haploinsufficient) or slightly favored (haploproficient), can be detected [10-13]. In our experiment, the haploinsufficient and haploproficient phenotypes detected in the presence of the N-BPs reveal alleles whose gene products are affected by the specific condition and, therefore, likely to be drug targets. With this approach we confirmed FPPS as the main *in vivo *target of N-BPs action and we identified additional biological processes affected by N-BPs, such as vacuolar acidification, microtubule dynamics, and DNA replication, underlying the complex cellular effects that bisphosphonates have on cells.

## Results

### Competition experiments

The wild type *S. cerevisiae *strain BY4743 was tested for its response to ALE, IBA and RIS in order to select a sub-lethal dose to use with the collection of deletion mutants. RIS and IBA were powerful growth inhibitors, whereas ALE had a much weaker effect on the yeast cells (Figure S1 in Additional data file 1). Competition experiments with 5,936 hemizygous yeast mutants were carried out in the presence of each drug. Strains showing a significant change in their growth rate were identified. The significance threshold was chosen to give a false discovery rate of q < 0.001 for the haploinsufficient strains, and of q < 0.01 for the haploproficient ones since only a smaller number of strains displayed an increase in growth rate (see Materials and methods; Additional data files 2, 3, 4 and 5). Some strains (197 for RIS, 250 for ALE and 283 for IBA) were so compromised by N-BPs that they disappeared from the population after 10 to 12 generations (Additional data file 2). These strains are referred to as quick disappearing (QD) and, for such mutants, there are no 'growth rate difference' values.

**Figure 1:**
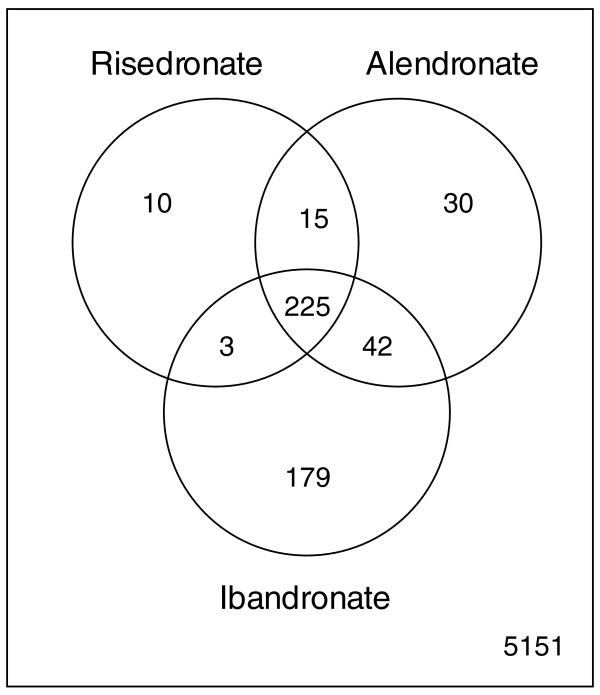
Venn diagram of numbers of haploinsufficient and haploproficient genes after removal of bad tags and dubious ORFs. Haploinsufficient genes are often shared between all three drug conditions; genes involved in heat shock response show a similar phenotype. IBA and ALE appear to have an overlapping mode of action on genes associated with secondary N-BP targets, such as chromatin structure, but not on primary, mevalonate-dependent interactions, while RIS and IBA share the main N-BP target, the farnesyl transferase ERG20, part of the mevalonate pathway.

Lists of strains showing haploinsufficient and haploproficient profiles in the presence of the drugs are shown in Additional data file 2 and Additional data file 3, respectively. From these lists we subsequently removed the strains that carried a mutation in a dubious ORF (according to the *Saccharomyces *Genome Database [14]), those known to harbor erroneous TAGs [15] and those showing a slow growing phenotype on a minimal medium (according to the *Saccharomyces *Genome Database), since their haploinsufficiency could depend on the nutrient limiting-medium rather than on the specific drug. Lists of haploinsufficient and haploproficient strains after the filtering process are given in Additional data files 4 and 5, respectively. About 45% of the haploinsufficient strains (including the QD) overlapped across the three conditions (Figure 1) and there is a common fingerprint when strain growth rates are compared between the three conditions (Figure S2 of the Additional data file 1).

The highest numbers of haploinsufficient and haploproficient genes were scored in the presence of IBA. The sensitivity and reliability of the 'barcode' method were demonstrated by the severe haploinsufficiency, in the presence of RIS and IBA, of the gene *YJL167W*, which encodes the yeast farnesyl pyrophosphate synthetase Erg20p, the only known molecular target of N-BPs in humans [16]. Interestingly, ALE, which had only a very weak effect on *S. cerevisiae *(Figure S1 in Additional data file 1), does not seem to compromise the growth rate of a *YJL167W *hemizygous mutant, suggesting that its interaction with FPPS is limited or inefficient in yeast. Gene Ontology analysis applied to the data showed enrichment in categories such as chromatin remodeling and, more generally, DNA packaging. A detailed analysis of their human orthologs revealed the presence of several genes encoding components of a complex that responds to DNA damage [17], including *SMARCB1 *(yeast *YLR321C*), *MCM5 *(yeast *YLR274W*), *MCM6 *(yeast *YGL201C*) and *DBF4 *(yeast *YDR052C*). In particular, *DBF4 *was found to be haploproficient in the presence of IBA. This was confirmed by growing separately both a hemizygote *DBF4 *mutant and the wild-type strain in the presence and absence of IBA. The results showed that the *DBF4 *mutant presents a quantitatively significant increase in final biomass (*P *< 6.4 × 10^-6^; Additional data file 6), suggesting that such a hemizygous mutant can partially counterbalance the N-BP's toxicity.

Among the strains showing a marked haploinsufficient profile in the presence of the three drugs, we found genes related to proton pumps, which were suggested as N-BP targets before the discovery of FPPS's involvement [18]. Several regulators of the plasma membrane H^+^-ATPase pump PMA1 (encoded by *YGL008C *(*PMA1*)) were highly haploinsufficient: *YDR033W *(*MRH1*; of unknown function), QD in all three conditions, may be involved in PMA1 regulation according to its similarity to HSP30 [19]; *YBL069W *(*AST1*), which plays a role in targeting Pma1p to the membrane [20], is also haploinsufficient in all three conditions; and *YCR024C-A *(*PMP1*), which encodes a regulatory subunit of PMA1 [21], is severely haploinsufficient in the presence of IBA. No human ortholog of *MRH1 *has been found, although this does not exclude the possibility of a functional homolog that could represent a new effector of N-BPs. Several proteins whose functions are linked to microtubules were also significantly affected by the treatments. The strains hemizygous for *ATG11 *(*YPR049C*), *ATG14 *(*YLR295C*) and *ATG15 *(*YCR068W*), whose gene products are involved in autophagy and vacuolar processing, display a haploinsufficient profile in at least one of the drug conditions used. Moreover, the deletion mutant for *ATG4 *(*YNL223W*), haploinsufficient in the presence of RIS, encodes a mediator for the attachment of autophagosomes to microtubules via its interaction with Tub1p and Tub2p and has a human homolog, *ATG4B*. The hemizygous mutants for alpha-tubulin (*TUB3*), ADP ribosylation factor (*ARF1*) and alpha-tubulin folding protein (*ALF1*) also show clear haploinsufficient profiles. In particular, the growth disadvantage of *ALF1 *mutant (*YNL148C*), homologous to the mammalian tubulin cofactor B gene (*TBCB*), was confirmed by growing individually both the hemizygote mutant and the wild-type strain in the presence of IBA (quantitatively significant decrease of final biomass yield, *P *< 0.0043; Additional data file 6).

About 135 strains were haploproficient (q < 0.01), and the most marked phenotypes were those related to the internalization of molecules. For example, *RAV1 *(*YJR033C*) encodes one of the subunits of the RAVE complex responsible for the assembly of the yeast V-ATPase and vacuolar acidification. These data indicate that a defect in either the assembly of the RAVE complex or in the acidification of the vesicles confers an advantage to the cell in the presence of N-BPs (its human homolog encodes DmX-like 1 protein). Overall, our data strongly suggest the involvement of other effectors, besides FPPS, in N-BP-induced toxicity. The human homologs of the haploinsufficient and haploproficient genes were studied in human cell lines to see whether they display similar functions. In particular, since we could identify DNA damage and cytoskeleton dynamics as the novel processes affected by N-BP treatment, we focused our attention on genes that constitute fundamental nodes in these processes.

### N-BPs induce DNA damage, modulate *DBF4 *expression and trafficking and induce cell cycle arrest

**Figure 2:**
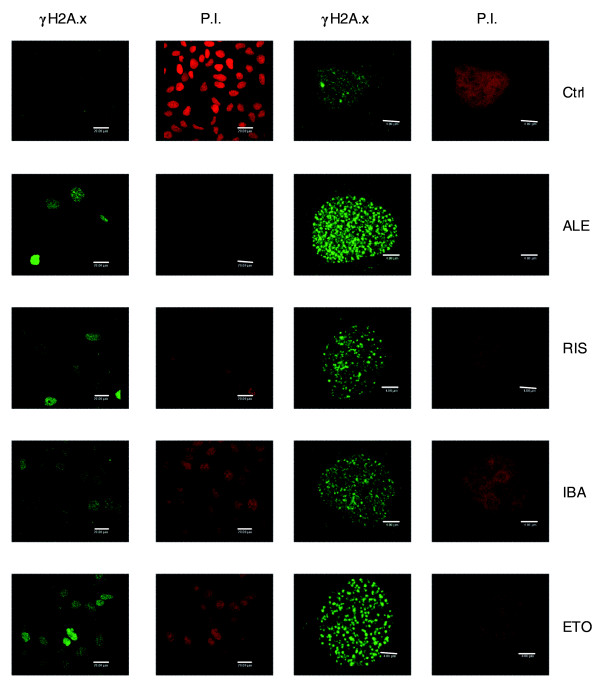
N-BPs induce DNA double-strand breaks. MCF-7 cells were treated with 10^-4^M ALE, IBA and RIS for 72 h. As a positive control, the cells were treated with 50 μM etoposide (ETO) for 24 h. Cells were then fixed and stained for γH2A.x (green). Nuclei were visualized by propidium iodide (P.I.) counterstaining (red). Scale bar: 4 or 20 μm.

N-BP-induced toxicity in *S. cerevisiae *suggested the possible involvement of a group of human gene orthologs to those involved in yeast fitness variation and connected to DNA damage: *SMARCB1*, *MCM5*, *MCM6 *and *DBF4*. Since evidence of DNA damage upon N-BP treatment has been reported after treatment with zoledronic acid [22,23], we chose to evaluate the formation of DNA double strand breaks in the presence of ALE, IBA and RIS by measuring the phosphorylation status of the histone variant H2A.x (that is, γH2A.x) [24]. Immunofluorescence microscopy, performed on MCF-7 cells using a specific antibody directed against γH2A.x revealed the formation of positive double strand break foci after treatment with the three N-BPs for 72 h at 10^-4 ^M (Figure 2). The percentage of cells presenting γH2Ax foci, evaluated by counting the foci-positive cells on six different fields in three different experiments was 83 ± 15, 75 ± 14, 75 ± 9, and 98 ± 4 in ALE, RIS, IBA and etoposide treated cells, respectively.

**Figure 3:**
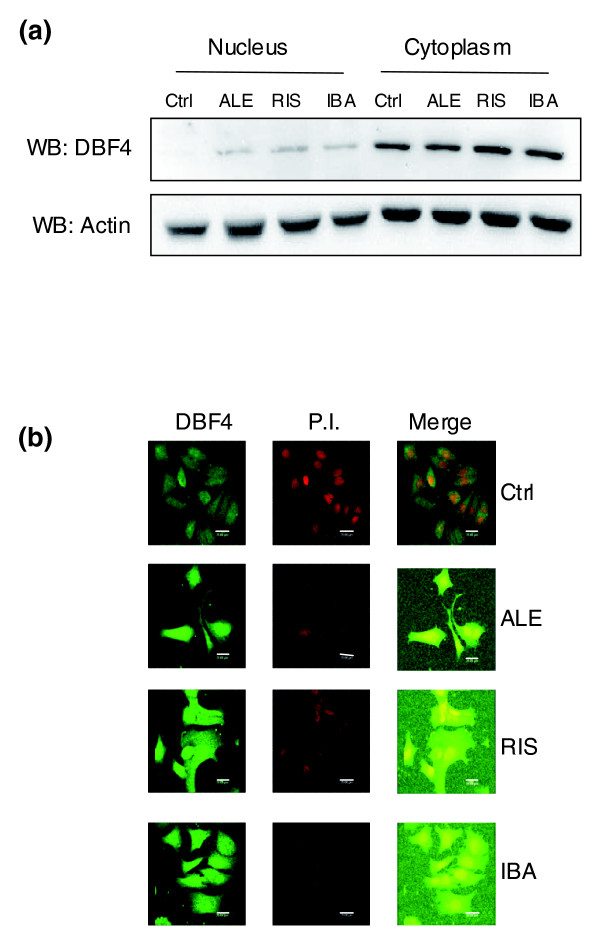
N-BPs modulate *DBF4 *expression and trafficking. MCF-7 cells were treated with 10^-4 ^M ALE, RIS and IBA for 72 h, and nuclear and cytoplasmic extracts were subjected to SDS-PAGE. **(a) **Representative western blot (WB) analysis of DBF4 expression level; actin was used as loading control (Ctrl). **(b) **MCF-7 cells were fixed and stained for DBF4 (green) after stimulation with 10^-4 ^M ALE, RIS or IBA for 48 h. Nuclei were visualized by propidium iodide (P.I.) counterstaining (red). Scale bar: 20 μm.

DBF4 is a well known S-phase checkpoint effector [25], and the DBF4-Cdc7 complex is crucial for the initiation of the DNA replication by activating the minichromosome maintenance (MCM) protein. Both DBF4-Cdc7 and MCM proteins are phosphorylated by the protein kinases ATM and ATR [25]. Cells that are hemizygous for *DBF4 *are severely haploinsufficient; however, this study shows that such a disadvantage is compensated for by the presence of N-BPs, suggesting the occurrence of epistatic interactions involving the *DBF4 *gene. Since DBF4 protein accumulates in the nuclei of G_1_-, S-, and M-phase-arrested cells [25], we decided to follow its localization upon N-BP stimulation via immunoblot analysis of nuclear and cytoplasmic extracts of MCF-7 cells. Upon stimulation with ALE, RIS and IBA, DBF4 protein accumulates within the nuclear compartment (Figure 3a). These data have also been confirmed by immunofluorescence experiments through confocal analysis, where the presence of DBF4 in the nuclei of cells treated with N-BPs is particularly evident in the merged picture (Figure 3b). Interestingly, DBF4 appeared to have a molecular weight of about 118 kDa, instead of the nominal 77 kDa, suggesting that a hyperphosphorylated form of the protein was present in the cell. This has been confirmed by phosphatase treatment experiments (data not shown). Flow cytometry analysis after 72 h of 10^-4 ^M N-BP treatment showed that the drugs were able to block the cell cycle of MCF-7 cells in the S-phase (Figure S3a in Additional data file 1). In particular, the number of cycling cells in the S-phase increased from 16% to 21% for IBA, to 28% for RIS and to 38% for ALE. This observation was concomitant with a reduction of cells in the G_0_/G_1 _phase: 78% in control cells versus 75%, 64% and 60% in IBA, RIS and ALE treated cells, respectively. Moreover, the same treatment led to an increase in the amount of dead cells in the sub-G_0_/G_1 _phase from 13% to 17%, 49% and 58% for IBA, RIS and ALE, respectively (Figure S3b in Additional data file 1). Notably, the three drugs showed different potency, with ALE being the more active both in cell-cycle arrest and in the induction of cellular death.

### *DBF4 *down-regulation leads to protection from N-BP toxicity in MCF-7 cells

**Figure 4:**
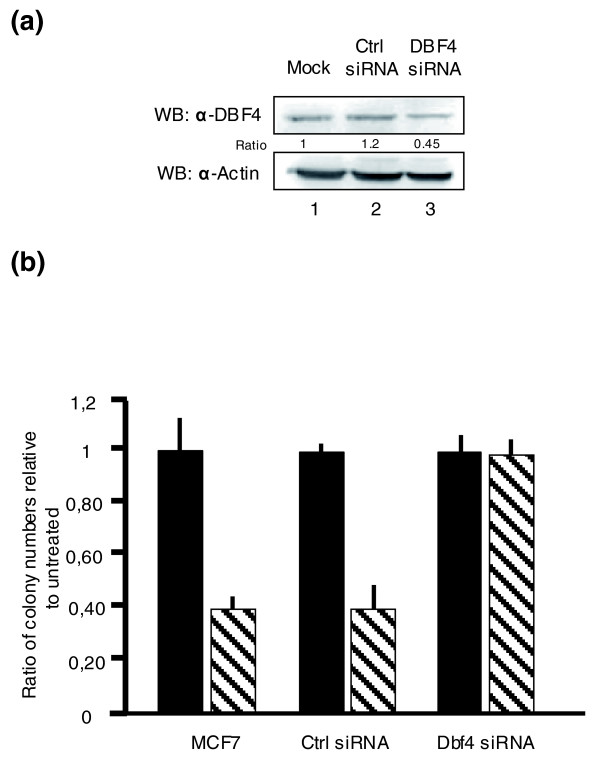
Effect of ALE on the clonogenic growth of *DBF4*-downregulated MCF-7 cells. **(a) **Endogenous *DBF4 *protein was downregulated by siRNA. MCF-7 cells were transfected with only oligofectamine (lane 1, mock), 40 nM control siRNA Luciferase GL2 Duplex (lane 2), and 40 nM of siGENOME duplex pool directed against DBF4 (lane 3). The total protein extracts were subjected to SDS-PAGE and DBF4 protein levels were quantified by western blotting (WB) and actin was measured as loading control. Five hours after siRNA transfection, MCF-7 cells were subjected to ALE treatment at a concentration of 10^-6 ^M for 48 h. **(b) **Following stimulation, 1,000 cells were plated for the clonogenic assay. After 10 days, the colonies were stained with 10% crystal violet and scored using ImageQuant TL computer software. The experiments were performed in triplicates and the error bars represent standard error of the mean. Black bars represent untreated cells, while stripped bars correspond to *DBF4*-downregulated cells. Ctrl, control.

As the *DBF4 *hemizygous yeast strain showed a haploproficient behavior, the role of its mammalian ortholog *DBF4 *in the MCF-7 system was studied by reproducing the conditions present in the yeast fitness assay. DBF4 protein levels were down-regulated to about 50% of the normal expression by using small interfering RNA (siRNA; Figure 4a). The clonogenic assay showed that mock and control siRNA-transfected MCF-7 cells, when treated with ALE, displayed a significant reduction in colony formation in comparison with the untreated ones. In contrast, colony formation in *DBF4*-downregulated cells was similar to that of the untreated control, suggesting protection from the ALE-induced toxicity (Figure 4b).

### N-BP effects on microtubules organization and dynamics

**Figure 5:**
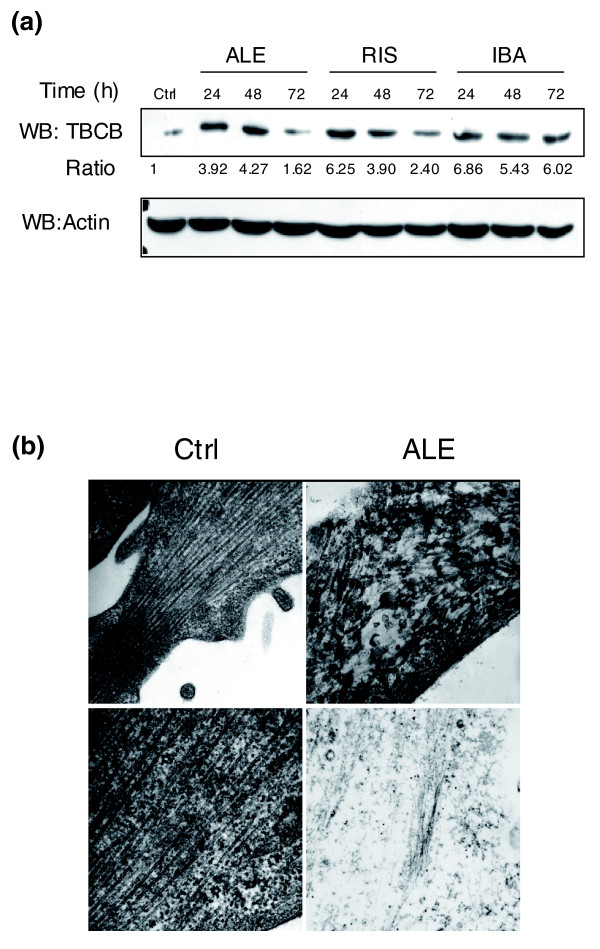
Effect of N-BPs on microtubule structure. **(a) **Effect of N-BP treatment on TBCB expression levels. Western blotting (WB) analysis showing the protein levels of TBCB after stimulation with 10^-4^M ALE, RIS and IBA for 24, 48 and 72 h, respectively. The signal given by total actin was used as a loading control (Ctrl). **(b) **N-BPs disrupt microtubule cytoskeleton organization. Ultrastructural pictures of MCF-7 cells under different conditions. Left panels: presence of a tightly packed bundle of microtubules arranged in a parallel way within a lamellipodial protrusion, under basal conditions (original magnifications: × 35,000 (top); × 45,000 (bottom)). Top right panel: irregular microtubular organization after N-BP treatment (10^-4 ^M, 72 h; original magnification × 35,000). Bottom right panel: anti-tubulin immunogold labeling of filamentous structures after N-BP treatment (original magnification × 22,000).

A group of genes associated with microtubule dynamics showed a haploinsufficient profile in yeast in the presence of N-BPs. Among these was *ALF1*, a homolog of the mammalian tubulin cofactor B (*TBCB*) gene, which encodes the α-tubulin folding protein. It has been demonstrated that changes in *TBCB *levels have a strong effect on microtubule growth. In particular, a recent paper reported that overexpression of *TBCB *can lead to microtubule depolymerization in growing neurites [26]. We therefore evaluated if N-BPs were able to modify TBCB protein levels in MCF-7 cells. Western blots were performed on total protein extracts from cells treated with high doses of N-BPs (10^-4 ^M) for 24, 48 and 72 h, using a specific antibody directed against TBCB. All three N-BPs used were able to increase TBCB protein levels and each showed a peculiar trend of induction, with ALE peaking at 48 h after stimulation, and RIS and IBA at 24 h after stimulation (Figure 5a). Electron microscopy on MCF-7 cells showed a marked effect of N-BPs on protrusions and lamellipodia/filopodia, where the parallel organization of the microtubules was replaced by a totally irregular one (Figure 5b). Under basal conditions, the MCF-7 cell cytoplasm showed a system of regularly arranged microtubules running parallel to each other, with close bundle formation at the level of lamellipodial protrusions (Figure 5b, top panels). After ALE treatment, dramatic tubulin involvement was evident since microtubules were markedly reduced in number and showed structural alterations such as irregularly wavy course and abrupt breakdowns (Figure 5b, top right). In addition, the concurrent presence of a lot of filamentous structures together with decoration by colloidal gold particles detectable after anti-tubulin antibody immunogold labeling (Figure 5b, bottom right) was visible. This completely new finding could be correlated to the effect that N-BPs have on TBCB. Preliminary experiments with nocodazole [27] suggested that N-BPs may affect microtubule dynamics (data not shown).

### N-BP treatment inhibits cell migration

Based on the finding that N-BPs may have an effect on tubulin dynamics, which is involved in many essential functions, including cell movement, we wondered whether N-BP treatment could disturb cell migration. As shown by the time-lapse microscopy analyses (Figure S4 in Additional data file 1), while IBA seemed to have only a slight effect, both ALE and, to higher extent, RIS blocked the migration of MCF-7 cells.

### DBF4 and TBCB are differently rescued by geranylgeranyl pyrophosphate

**Figure 6:**
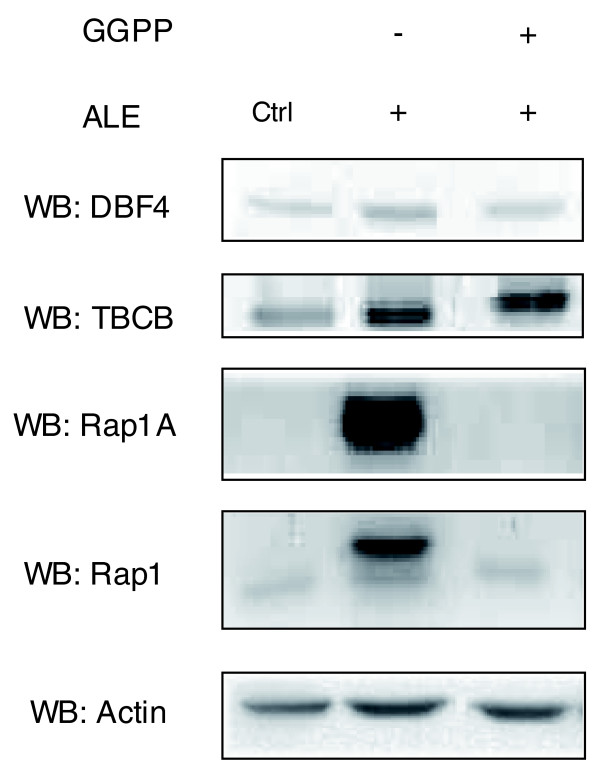
N-BP-induced accumulation of unprenylated Rap1A and increase of DBF4, but not of TBCB, can be reversed by GGPP. Western blot (WB) analysis of MCF-7 cells treated with 10^-4 ^M ALE alone or in combination with 25 μM GGPP. The same volume of absolute ethanol was used as control vehicle of GGPP (Ctrl). Actin was used to show equal loading of the lanes.

The main mechanism of action through which N-BPs block osteoclast-mediated bone resorption is via FPPS inhibition of the mevalonate pathway [28]. It has been previously shown that these drugs inhibit the growth of various cancer cell lines through a similar mechanism [29,30]. To assess the contribution of FPPS inhibition on the increase of DBF4 and TBCB protein levels, we performed rescue experiments with geranylgeranyl pyrophosphate (GGPP) in MCF-7 cells. Cells were grown with 10^-4 ^M ALE for 48 h and the accumulation of unprenylated Rap1A was used as a marker for the inhibition of the pathway [31]. ALE induced an increase in the accumulation of unprenylated Rap1A that was reversed by simultaneous addition of 25 μM GGPP (Figure 6). Interestingly, while the ALE-induced increase of DBF4 was reversed by simultaneous addition of 25 μM of GGPP, the increase in TBCB remained unaffected by GGPP treatment, suggesting that different pathways are involved in the N-BP-induced upregulation of DBF4 (mevalonate-dependent) and TBCB (mevalonate-independent).

## Discussion

N-BPs are potent inhibitors of osteoclast-mediated bone resorption and are used to relieve bone pain and to prevent skeletal complications in bone metastasis, most common in breast and prostate cancer [1]. Furthermore, several *in vitro *and *in vivo *studies have reported the ability of N-BPs to exert a direct anti-tumor effect on cancer cell lines [28]. The actions of N-BPs on tumor cell lines include the promotion of apoptosis and the inhibition of cellular adhesion and invasion [29,30,32]. However, besides the established inhibition of protein prenylation [16], little is known about other potential mechanisms involved in N-BP-induced toxicity. In recent years, with the emerging field of chemogenomics, several large scale efforts have been made to efficiently identify new therapeutic targets. In this work we used the 'haploinsufficiency profiling approach', pioneered in yeast by Giaever and co-workers [7], in order to identify secondary targets of N-BPs. *S. cerevisiae *is very versatile and easily managed and several high-throughput tools are in place for this it [8]. Moreover, over 30% of human genes involved in diseases have a homolog in yeast [33], making it an ideal experimental system to open new promising perspectives for translational medicine. We carried out a series of competition experiments with a barcoded collection of 5,936 hemizygous mutants [4,5] in the presence of ALE, IBA and RIS in order to identify potential drug targets and gain insight into the molecular changes occurring in cells exposed to such drugs.

Interestingly, from our study it emerged that several different molecular players contribute to N-BP-induced toxicity, suggesting that, besides FPPS, which is the primary enzymatic target and was confirmed by our analysis, there are other molecules whose functions or expression levels are altered by the treatment. Moreover, these effectors could help in defining the exact mechanisms at the root of the different degrees of potency observed with each N-BP. Notably, some of the targets we found have already been proposed as molecules affected by N-BPs. First, in the presence of all three drugs, the most compromised yeast strains in the competition experiment were the hemizygous mutants for *MRH1 *and *AST1*, which are related to ATPase-proton pumps. *MRH1*, as a homolog of *HSP30*, has a putative function in the regulation of the expression of the plasma membrane H^+^-ATPase pump, *PMA1 *[19], while *AST1 *is responsible for its correct targeting onto the cell membrane [20]. Furthermore, the product of *PMP1*, a small single-span membrane protein that regulates the H^+^-ATPase pump [21], was also haploinsufficient with IBA. Interestingly, the *PMA1 *hemizygous mutant itself shows no significant haploinsufficient phenotype, suggesting that the regulation of this gene, rather than its genome copy number, is responsible for the pharmacological effects of the N-BPs. Other genes emerged as a consequence of their involvement in N-BP uptake or internalization. As an example, we found that the *RAV1 *hemizygous mutant strain is haploproficient when grown in the presence of N-BPs. *RAV1 *belongs to the RAVE complex, which is responsible for vacuolar acidification via the V-ATPase. Recent experiments in osteoclast cell lines have shown that N-BPs are internalized via endocytosis and that endosomal acidification is required for their translocation into the cytoplasm [31]. Our data support this hypothesis; in fact, a deficiency in the acidification of the endocytic vesicles preventing the release of the N-BPs into the cytosol would confer a growth advantage on the cell in the presence of the drugs.

The 'barcode' technology allowed us to identify two novel biological processes that appeared to be particularly affected by the treatments: DNA damage and cytoskeleton dynamics. DNA damage has been suggested in earlier studies as the cause of the activation of *ATM *and *ATR *after zoledronic acid stimulation, but clear evidence was still missing for other N-BPs with different side chains [22,23]. We have demonstrated for the first time that, in MCF-7 cells, IBA, RIS and ALE are able to cause a significant accumulation of double strand breaks. Among the DNA damage-related genes that emerged from our analysis, we found that encoding the regulatory subunit of the DBF4-Cdc7 complex, which is involved in DNA replication. In our mammalian model, DNA damage is followed by DBF4 phosphorylation and nuclear translocation, events that we hypothesized to be the triggers of cell cycle arrest observed in S-phase. Moreover, *DBF4 *seems to be a key player in the mechanisms of N-BPs toxicity, since its downregulation protected the cells from the anti-proliferative effect exerted by the N-BPs. In general, this finding opens the possibility that reverting to a haploproficient phenotype may constitute a mechanism by which cells become resistant to N-BPs. The second detected mechanism related to the N-BPs' effects is microtubules dynamics. In particular, we identified *ALF1*, a regulator of alpha-tubulin folding, whose human homolog is *TBCB*, as the most interesting gene. In MCF-7 cells, we observed a significant upregulation of TBCB protein levels after N-BP treatment and the simultaneous loss of microtubule architecture in sites of active microtubule assembly, such as protrusions. Therefore, *TBCB *upregulation represents a novel mechanism through which N-BPs could affect cellular viability, and further experiments will be performed to define the effects of N-BPs on microtubule-related processes, such as mitotic spindle formation and vesicular transport.

## Conclusions

This study has exploited the heterozygous yeast mutant collection for mode-of-action discovery of secondary targets of N-BPs, the elected drugs for the treatment of bone resorption and cancer-induced bone diseases [1,34]. In particular, this work allowed the discovery of two novel biological processes involved in the cytotoxic effects of the N-BPs, DNA damage and microtubule assembly, and, thanks to the 'barcode' approach, these could be linked directly to the responsible genes, *DBF4 *and *TBCB*. In this case, a strong conservation between yeast and mammalian targets was seen, since their involvement was confirmed also in our human breast cancer cell line, MCF-7, used as a mammalian model. Neither *DBF4 *nor *TBCB *have been described before as N-BP targets, and these findings may open up new opportunities for the development of new compounds with antitumor activity.

## Materials and methods

### Chemicals

All the chemicals were from Sigma Aldrich Co. (Milan, Italy) unless otherwise specified. The GGPP was from American Radiochemicals Inc. (St Louis, MO, USA), and the bisphosphonates were provided by Procter and Gamble Pharmaceuticals (Cincinnati, OH, USA).

### Yeast strains and cell lines

The yeast strains used in this work are BY4741 (*MAT*a, *his3Δ1*, *leu2Δ0*, *met15Δ0*, *ura3Δ0*), and BY4742 (*MATα*, *his3Δ1*, *leu2Δ0*, *lys2Δ0*, *ura3Δ0*) and BY4743 (*MAT*a/*MATα his3Δ1*/*his3Δ1 leu2Δ0/leu2Δ0 met15Δ0*/*MET15 LYS2*/lys2Δ0 *ura3Δ0*/*ura3Δ0*). The hemizygous deletion collection, in the diploid BY4743 background, was obtained from the *Saccharomyces *Deletion Consortium [35].

The human breast adenocarcinoma MCF-7 cell line was obtained from the ATCC collection (Manassas, VA, USA), and cultured in DMEM. All the yeast media, YPD, SD and F1, were prepared as described previously [10,36,37]. The hemizygous deletion pool was created manually by growing the strains in YPD with 15% (v/v) glycerol using 96-well plates, at 30°C until they reached a stationary phase (48 h). Using a multi-channel pipette, the mutant strains were combined together in a sterile Petri dish, before being transferred to a 50 ml Falcon tube. The pool was stored at -80°C in 1 ml aliquots.

### Competition experiments

To determine the sub-lethal concentrations of the N-BPs, different concentrations of RIS, ALE and IBA were added to cultures of BY4741 and BY4743 grown in F1 medium.

An aliquot (10^7 ^cells) of the hemizygote pool was inoculated into flasks containing 20 ml of YPD medium and allowed to grow in batch for 18 h at 30°C, with shaking at 170 rpm. The cells were then diluted to an OD_600 _of 0.005 in 10 ml of F1 medium containing 5 × 10^-4 ^M RIS, 5 × 10^-3 ^M ALE or 5 × 10^-4 ^M IBA. To maintain exponential growth, the cells were allowed to grow for six generations before being diluted back to an OD_600 _of 0.02 in fresh F1 medium containing the drugs. Samples of the cultures were taken throughout the experiment, in particular at the beginning of the competition, just before adding the drugs (generation 0) and after 10 to 12 and 17 to 20 generations.

### Hybridization and statistical analysis

The DNA was extracted from the samples using a DNA tissue kit (Qiagen, Crawly, West Sussex, UK). The concentration of the genomic DNA was determined using a Nanodrop (Agilent, West Lothian, UK) device. The amplifications of the tags and the hybridization protocol were carried out as described [4]. The arrays were normalized by median centering intensity values from tags corresponding to mutants, as described [10]. Briefly, log-ratios were calculated between the initial time point, G0, and subsequent time points, G10 and G20. This aimed to eliminate tag-specific biases and further normalized the data. Growth rates were estimated by robust linear regression on the normalized log-ratios. Type I error rates (*P*-values) were estimated by model-based resampling with suitably re-scaled residuals. False discovery rates (q-values) were estimated according to Benjamini and Hochberg [38]. A q-value lower <0.001 was set as threshold for a growth rate difference to be considered statistically significant for haploinsufficient genes, while q < 0.01 was set as the threshold for haploproficient genes. Gene Ontology analysis was carried out using GOMINER on filtered lists of genes [39].

### Growth of selected strains on a microplate reader

The strains YDR052C (*DBF4*) and YNL148C (*ALF1*) were re-tested singularly. Accurate growth measurements of the selected single mutants and the wild-ype parent (BY4743) in both the presence and absence of IBA were produced using a Microplate Reader (FLUOstar OPTIMA, BMG Labtech, Offenburg, Germany). The optical density measurement at 600 nm was taken every 2 minutes for a 24 h period. The maximum growth rate and final biomass yield were calculated according to Warringer and Blomberg [40]. Three biological replicates, each comprising three semi-technical replicates, were carried out for each mutant strain tested. Two way ANOVA was carried out for each deletion strain to determine if there was a significant interaction between the drug and the deletion strain when compared to the effect of the drug on the parental background.

### Cell cycle analysis

Subconfluent MCF-7 cultures (ATCC), grown in DMEM supplemented with 5% fetal bovine serum (Euroclone Ltd., Torquay, UK), 0.1 mM non-essential amino acids and 1 mM sodium pyruvate, were incubated in the presence or absence of 10^-4 ^M N-BPs for 72 h and harvested as reported in [41]. Cell cycle distribution was examined by flow cytometry, and data were analyzed with Cell Quest™ and ModFit LT (FACScan, Becton Dickinson, Franklin Lakes, NJ, USA).

### Preparation of protein extracts and western blot analysis

Cell nuclear extracts were prepared as described previously [42] and analyzed for protein content (Bio-Rad Protein Assay, Bio-Rad Laboratories, Muenchen, Germany). To prepare total protein extracts, cells were lysed in a mild buffer (1% NP-40, 150 mM NaCl, 10 mM Tris, 2 mM EDTA, pH 7.2); the suspension was then incubated at 4°C for 20 minutes and then subjected to centrifugation for 20 minutes at 12,000 ×g; the supernatant was collected and transferred to a new tube as total extract.

The cellular extracts were electrophoresed and then transferred to nitrocellulose membranes as previously described [42]. Blots were incubated with the following polyclonal antibodies: rabbit anti-Dbf4 (Santa Cruz Biotechnology Inc., Santa Cruz, CA, USA), rabbit anti-actin (Sigma), goat anti-Rap1A (C-17 - epitope mapping at the C-terminus of Rap 1A of human origin), and rabbit anti-Rap1 (121 - epitope mapping near the C-terminus of Rap 1 of human origin) (Santa Cruz Biotechnology), and anti-TBCB, a generous gift of JC Zabala, Universidad de Cantabria, Santander, Spain. The blots were then incubated with the corresponding peroxidase-conjugated anti-serum (Sigma). The bands were quantified as reported in [41].

### Immunofluorescence and confocal microscopy studies

For γH2A.x detection, cells were seeded on slides and the next day treated with 50 μM etoposide for 24 h (positive control), 10^-4 ^M N-BPs for 72 h or phosphate-buffered saline (control). Cells were then fixed, blocked and permeabilized as reported in [41] and incubated with the monoclonal antibody anti-γH2A.x (clone JBW301, Upstate, Lake Placid, NY, USA) for 2 h. After washing, they were incubated with the secondary antibody Alexa Fluor 488-conjugated (Molecular Probes Inc., Eugene, OR, USA) for 90 minutes. Nuclei were visualized by 1 μg/ml propidium iodide counterstaining.

For DBF4 detection, cells were treated without or with 10^-4 ^M N-BPs for 72 or 48 h, respectively. Cells were processed as described with polyclonal anti-Dbf4 antibody for 2 h. After washing, the cells were incubated with the secondary antibody Alexa Fluor 488-conjugated (Molecular Probes) for 90 minutes. Nuclei staining was performed as described above. The microscope slides were mounted and visualized through a Leica TCS SP laser-scanning confocal microscope [41].

### Time-lapse microscopy

Cells were cultured until reaching confluence, synchronized for 24 h in the absence of serum, than a wound was created by scraping the monolayer with a single-edge razor blade. The cells were then treated or not with 10^-4 ^M N-BPs. Cell migration was followed for the next 48 h (Leica AF6000 LX), taking phase-contrast photographs every 4 h.

### Electron microscopy and immunogold labeling

Subconfluent cultures of MCF-7 cells were incubated in the presence or absence of 10^-4 ^M ALE for 72 h. Cells were fixed in 4% glutaraldehyde in 0.1 M phosphate buffer, post-fixed with 2% OsO_4 _dissolved in the same buffer, and embedded in Epon 812 resin. Thin sections were collected on copper grids with 2 × 1 mm slots and contrasted with uranyl acetate and lead citrate. Observations were made using a Philips CM12 STEM transmission electron microscope. For immunogold labeling of tubulin, cells were fixed in neutral buffered 4% paraformaldehyde, dehydrated in graded ethanol and embedded in LR-White resin. Thin sections were collected on nickel grids, blocked with 5% normal goat serum, and incubated with 1:2,000 diluted mouse anti-tubulin monoclonal antibody, followed by diluted 18 nm gold-conjugated anti-mouse secondary antibody (Jackson ImmunoResearch Labs, Inc., Newmarket, England). After washing, sections were contrasted with uranyl acetate and lead citrate. As negative control, primary antibody was replaced with serum.

### RNA interference and clonogenic assay

Dbf4 expression was silenced by using the siGENOME duplex pool (Dbf4 catalog number MQ-004165-01) as reported [43] in MCF-7 cells. Control cells were transfected with control oligos (luciferase GL2 duplex, catalog number D001100-01-20). All the oligos were from Dharmacon Research Inc. (Lafayette, CO, USA). Transfection mixture was removed after 5 h and replaced with fresh medium containing 10^-6 ^M alendronate. After 48 h, cells were collected and counted and Dbf4 protein levels assessed by western blotting. For the Clonogenic Assay, 1,000 cells were plated in a 60 cm^2 ^petri dish in triplicate; after 10 days, the colonies were stained with crystal violet (10% w/v in ethanol 70%; Sigma) and counted using ImageQuant TL v2003.03 (GE Healthcare, Little Chalfont, Buckinghamshire, UK) with 50 cells being the requirement for scoring as a colony. Relative levels of cell survival were calculated by comparison with control without drug.

## Abbreviations

ALE: alendronate; DMEM: Dulbecco's modified Eagle's medium; FPPS: farnesyl pyrophosphate synthase; GGPP: geranylgeranyl pyrophosphate; IBA: ibandronate; N-BPs: nitrogen bisphosphonate; ORF: open reading frame; QD: quick disappearing; RIS: risedronate; siRNA: small interfering RNA; TBCB: tubulin cofactor B.

## Authors' contributions

DD and GT conceived the study and the experimental design. DD, GT, DCH, FQ and LM supervised the work. NB performed the genome-wide screen. FO and AB performed the electron microscopy analyses. MR and NB performed all other experiments. RH, IC and DCH, analyzed the data from the screens. DD, NB and GT wrote the paper.

## Additional data files

The following additional data are available with the online version of this paper: Figures S1, S2, S3 and S4 (Additional data file 1). A table listing haploinsufficient strains (q < 0.001; Additional data file 2). A table listing haploproficient strains (q < 0.01; Additional data file 3). A table listing haploinsufficient and QD strains after removal of bad tags (Additional data file 4). A table listing haploproficient strains after removal of bad tags (Additional data file 5). A table showing growth data and two-way ANOVA of the wild-type (WT) strain and the hemizygote mutants *DBF4 *(A) and *ALF1 *(B) in the presence and absence of the drug ibandronate (IBA) (Additional data file 6).

## Supplementary Material

Additional data file 1 Figure S1: wild-type S. *cerevisiae *responds to the N-BPs in a dose-dependent manner. All three drugs are able to inhibit growth and equimolar doses of each drug display a different degree of toxicity, with RIS and IBA being the most powerful. Representative growth curves of wild-type *S. cerevisiae *BY4743, grown in the presence of increasing concentrations of the indicated N-BPs for 20 h. Yeast growth was monitored using an OD reader with measurements every 5 minutes. Figure S2: N-BP sensitivity profiling for the significant strains. Each bar in the graph represents a specific strain; the more negative the value of the bar, the greater the rate of diminution of that strain from the pool. Figure S3: FACS analysis of the MCF-7 cell line treated with N-BPs. Percentage of cells in G_0_/G_1_, S and G_2_/M phases (A) after the exclusion of the sub-G_1 _population (dead cells), which was analyzed separately (B). Figure S4: effect of N-BPs on cell migration. MCF-7 cells were treated with or without 100 μM N-BP as described in Materials and methods and observed by time-lapse microscopy for the next 48 h, taking phase-contrast photographs every 4 h. The horizontal bars represent the limit of the slit performed on the cell monolayer at the start of the experiment. Five measurements per well were taken; the figure shows a representative experiment at 24 h and 48 h. Original magnification 200×. Click here for file

Additional data file 2 QD strains are shown in red. Click here for file

Additional data file 3 Haploproficient strains (q < 0.01). Click here for file

Additional data file 4 Haploinsufficient and QD strains after removal of bad tags. Click here for file

Additional data file 5 Haploproficient strains after removal of bad tags. Click here for file

Additional data file 6 Growth data and two-way ANOVA of the wild-type (WT) strain and the hemizygote mutants *DBF4 *(A) and *ALF1 *(B) in the presence and absence of the drug ibandronate (IBA). Click here for file
