# Supplementary material for: Identification of secondary targets of N-containing bisphosphonates in mammalian cells via parallel competition analysis of the barcoded yeast deletion collection
Source: Genome Biol. 2009 Sep 10;10(9):R93. doi: 10.1186/gb-2009-10-9-r93 (PMC2768982; doi:10.1186/gb-2009-10-9-r93)
Supplement: Additional data file 1 — Figure S1: wild-type S. cerevisiae responds to the N-BPs in a dose-dependent manner. All three drugs are able to inhibit growth and equimolar doses of each drug display a different degree of toxicity, with RIS and IBA being the most powerful. Representative growth curves of wild-type S. cerevisiae BY4743, grown in the presence of increasing concentrations of the indicated N-BPs for 20 h. Yeast growth was monitored using an OD reader with measurements every 5 minutes. Figure S2: N-BP sensitivity profiling for the significant strains. Each bar in the graph represents a specific strain; the more negative the value of the bar, the greater the rate of diminution of that strain from the pool. Figure S3: FACS analysis of the MCF-7 cell line treated with N-BPs. Percentage of cells in G0/G1, S and G2/M phases (A) after the exclusion of the sub-G1 population (dead cells), which was analyzed separately (B). Figure S4: effect of N-BPs on cell migration. MCF-7 cells were treated with or without 100 μM N-BP as described in Materials and methods and observed by time-lapse microscopy for the next 48 h, taking phase-contrast photographs every 4 h. The horizontal bars represent the limit of the slit performed on the cell monolayer at the start of the experiment. Five measurements per well were taken; the figure shows a representative experiment at 24 h and 48 h. Original magnification 200×. [file gb-2009-10-9-r93-S1.ppt]

## Slide 1
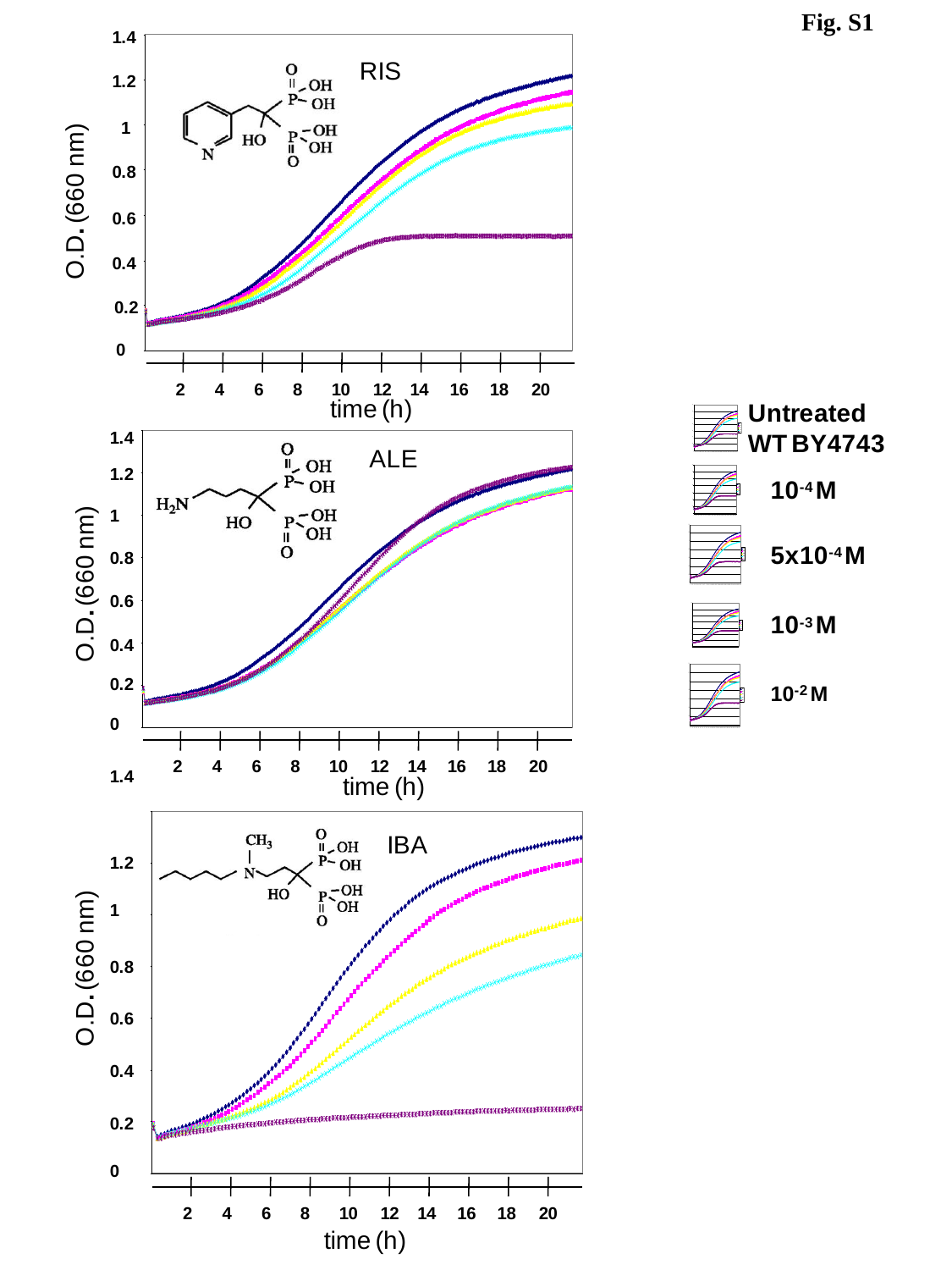

Fig. S1

## Slide 2
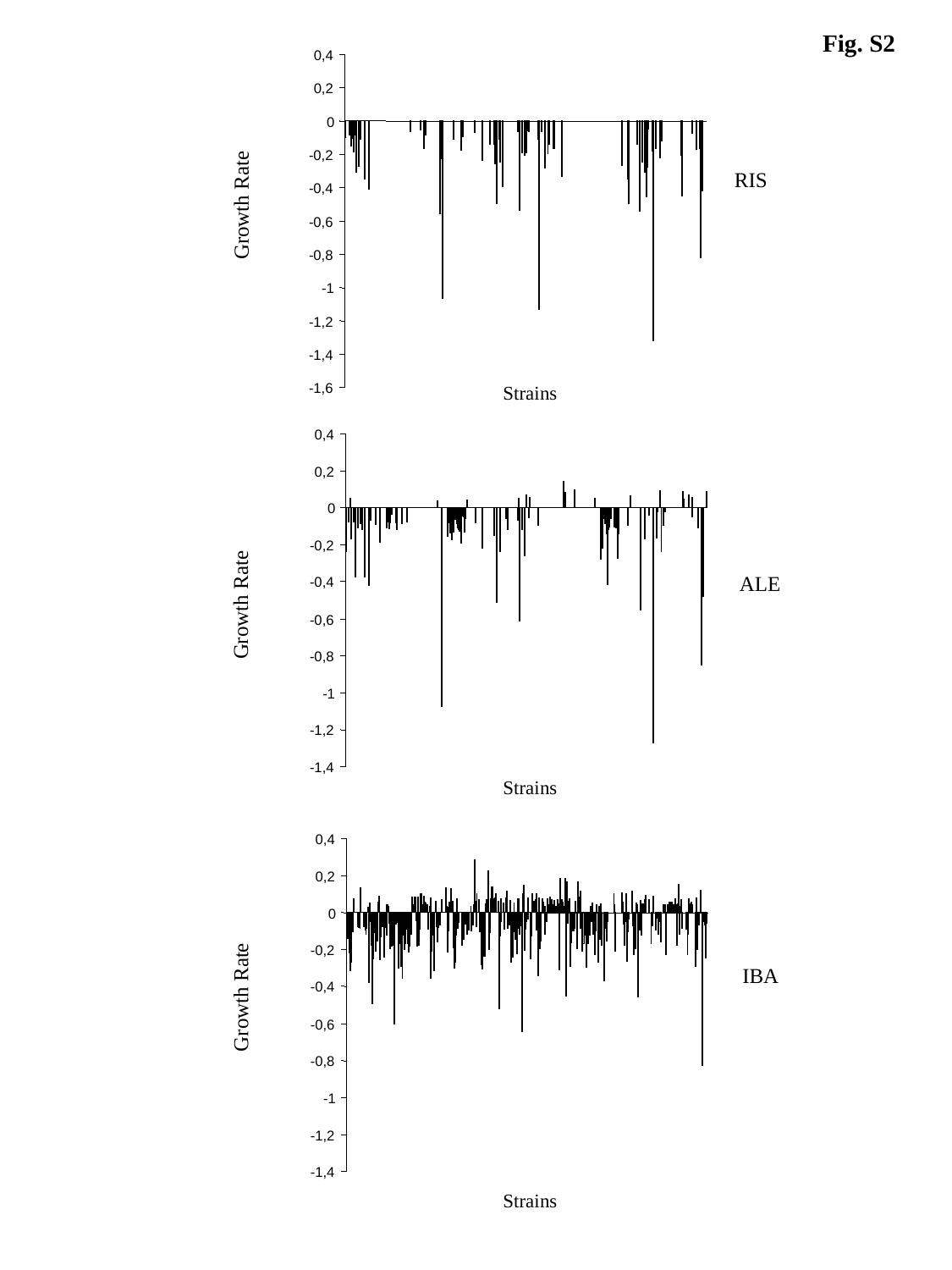

Fig. S2
Fig. S3
0,4
0,2
0
-0,2
-0,4
-0,6
-0,8
-1
-1,2
-1,4
-1,6
0,4
0,2
0
-0,2
-0,4
-0,6
-0,8
-1
-1,2
-1,4
0,4
0,2
0
-0,2
-0,4
-0,6
-0,8
-1
-1,2
-1,4
RIS
Growth Rate
Strains
ALE
Growth Rate
Strains
IBA
Growth Rate
Strains

## Slide 3
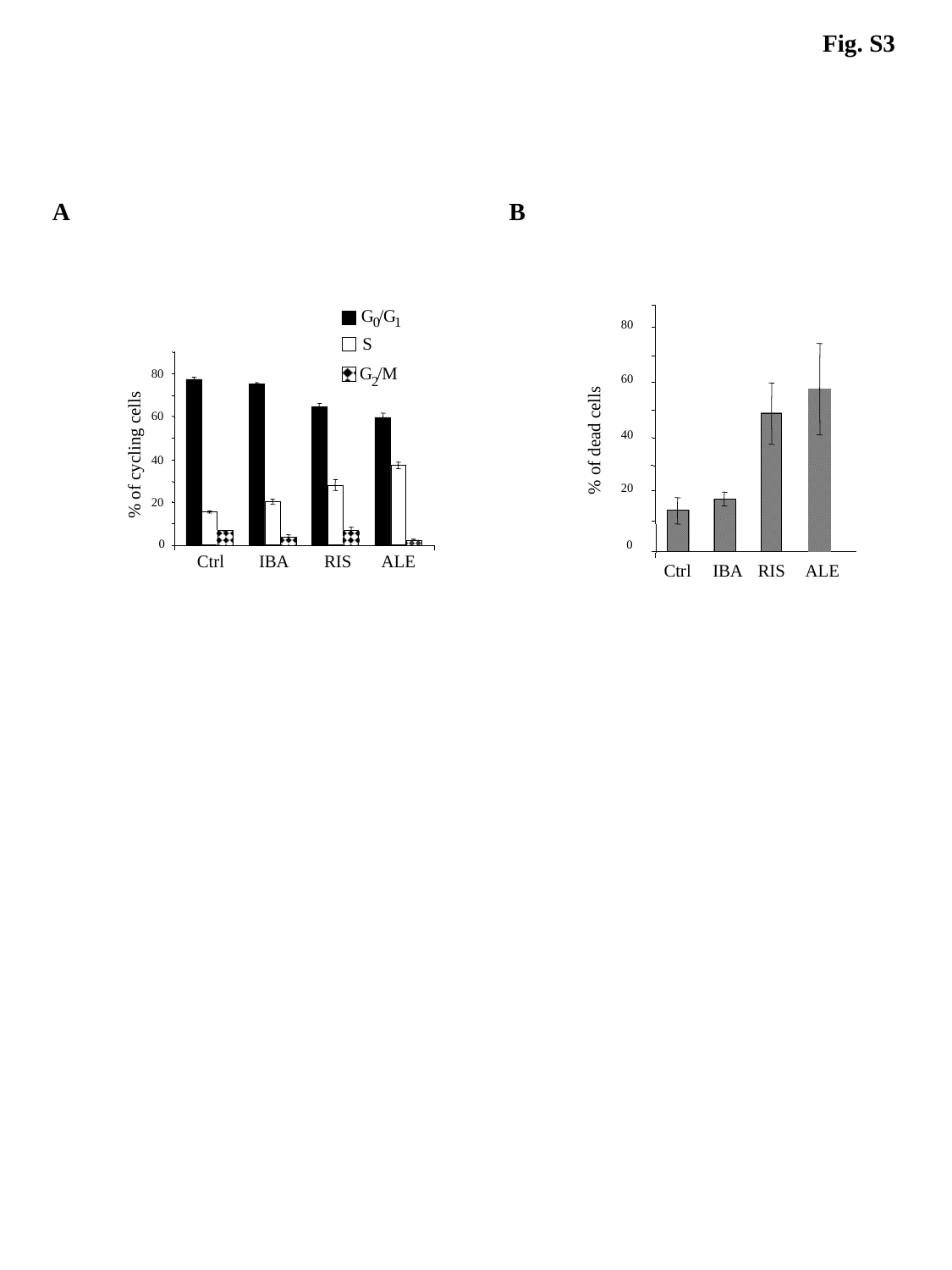

Fig. S3
A
B
G
/G
0
1
S
G
/M
2
80
60
% of cycling cells
40
20
0
Ctrl
IBA
RIS
ALE
80
60
40
% of dead cells
20
0
Ctrl
IBA
RIS
ALE

## Slide 4
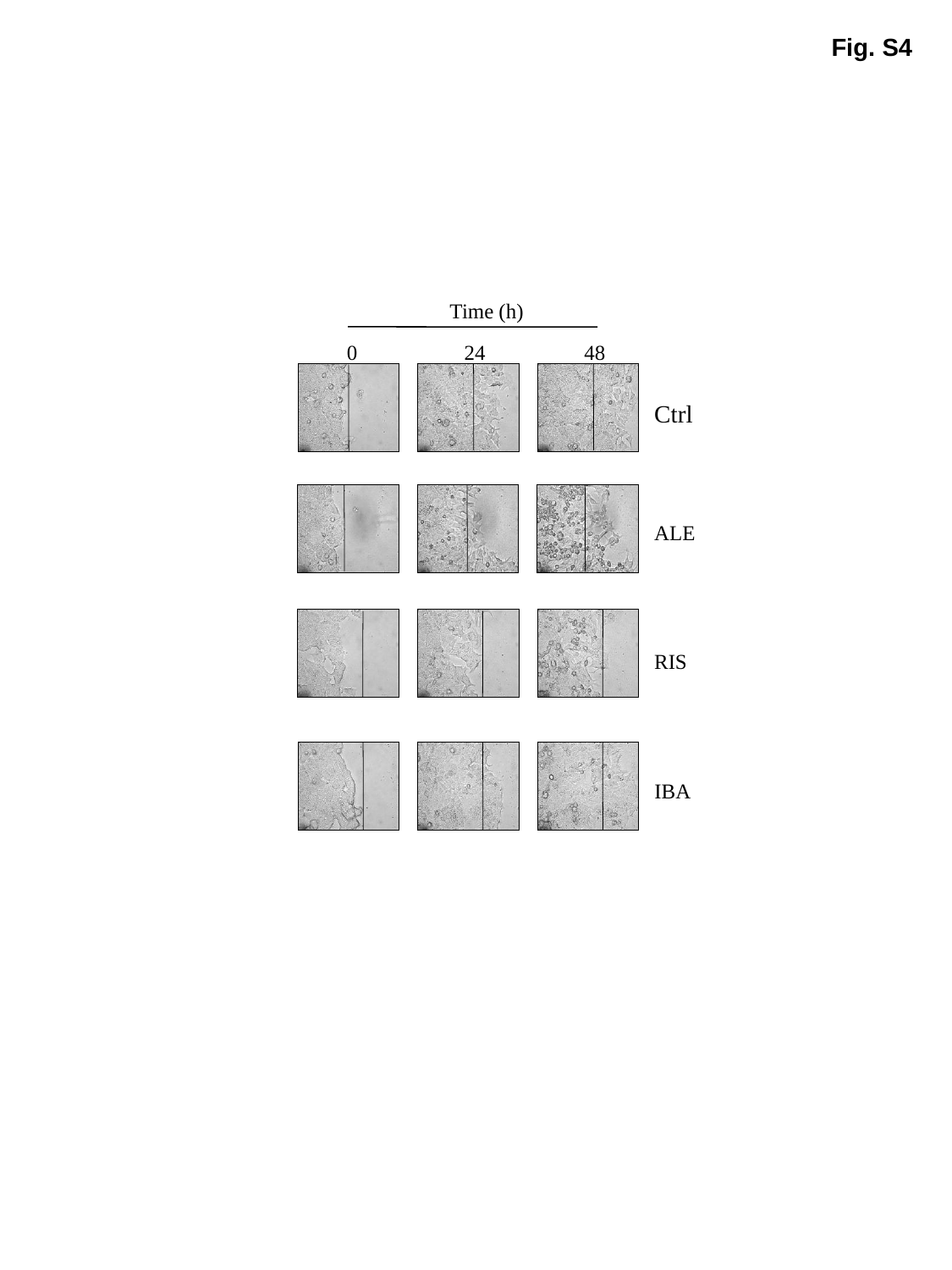

Fig. S4
Time (h)
0
24
48
Ctrl
ALE
RIS
IBA
