# Supplementary material for: Identification of secondary targets of N-containing bisphosphonates in mammalian cells via parallel competition analysis of the barcoded yeast deletion collection
Source: Genome Biol. 2009 Sep 10;10(9):R93. doi: 10.1186/gb-2009-10-9-r93 (PMC2768982; doi:10.1186/gb-2009-10-9-r93)
Supplement: Additional data file 6 — Growth data and two-way ANOVA of the wild-type (WT) strain and the hemizygote mutants DBF4 (A) and ALF1 (B) in the presence and absence of the drug ibandronate (IBA). [file gb-2009-10-9-r93-S6.ppt]

## Slide 1
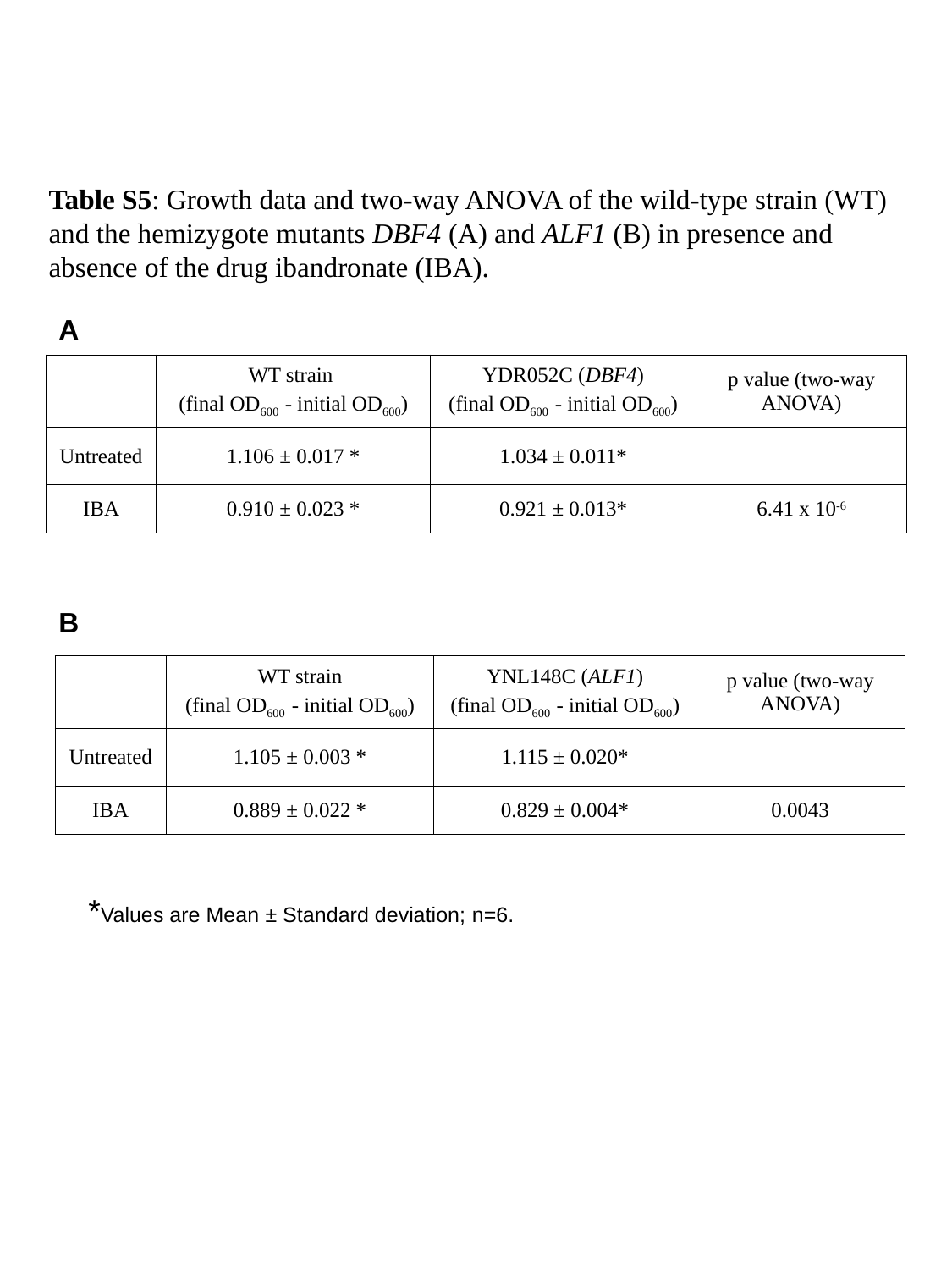

Table S5: Growth data and two-way ANOVA of the wild-type strain (WT) and the hemizygote mutants DBF4 (A) and ALF1 (B) in presence and absence of the drug ibandronate (IBA).
A
| | WT strain (final OD600 - initial OD600) | YDR052C (DBF4) (final OD600 - initial OD600) | p value (two-way ANOVA) |
| --- | --- | --- | --- |
| Untreated | 1.106 ± 0.017 \* | 1.034 ± 0.011\* | |
| IBA | 0.910 ± 0.023 \* | 0.921 ± 0.013\* | 6.41 x 10^-6^ |
B
| | WT strain (final OD600 - initial OD600) | YNL148C (ALF1) (final OD600 - initial OD600) | p value (two-way ANOVA) |
| --- | --- | --- | --- |
| Untreated | 1.105 ± 0.003 \* | 1.115 ± 0.020\* | |
| IBA | 0.889 ± 0.022 \* | 0.829 ± 0.004\* | 0.0043 |
*Values are Mean ± Standard deviation; n=6.
